# Diffusion-Informed Joint Segmentation Enhances Detection of Thalamic Atrophy in Parkinson’s Disease

**DOI:** 10.1007/s10548-026-01226-2

**Published:** 2026-06-30

**Authors:** Gozde Kizilates-Evin, Ani Kicik, Emel Erdogdu, Dilek Betul Saridede, Sevim Cengiz, Basar Bilgic, Hasmet A Hanagasi, Esin Ozturk-Isik, Tamer Demiralp, Ali Bayram

**Affiliations:** 1 Hulusi Behçet Life Sciences Research Laboratory, Neuroimaging Unit, Istanbul University, Istanbul, Türkiye; 2 Department of Physiology, Faculty of Medicine, Demiroglu Bilim University, Istanbul, Türkiye; 3 Department of Psychology, Faculty of Economics and Administrative Sciences, Isik University, Istanbul, Türkiye; 4 Health Institutes of Türkiye, Istanbul, Türkiye; 5 College of Technological Innovation, Zayed University, Dubai, UAE; 6 Behavioral Neurology and Movement Disorders Unit, Department of Neurology, Istanbul Faculty of Medicine, Istanbul University, Istanbul, Türkiye; 7 Institute of Biomedical Engineering, Boğaziçi University, Istanbul, Türkiye; 8 Department of Physiology, Istanbul Faculty of Medicine, Istanbul University, Istanbul, Türkiye; 9 Department of Neuroscience, Aziz Sancar Institute of Experimental Medicine, Istanbul University, Istanbul, Türkiye

**Keywords:** Thalamus, Thalamic nuclei, Segmentation, Magnetic resonance imaging, Diffusion tensor imaging, Mild cognitive impairment with Parkinson’s disease

## Abstract

The thalamus is a critical subcortical hub that relays sensorimotor information and regulates higher-order cognitive processes. Accurate delineation of thalamic nuclei is essential for elucidating disease mechanisms and tracking clinical progression. In this study, we compared two segmentation approaches implemented in FreeSurfer: the conventional structural method and a joint framework that integrates diffusion tensor imaging. Magnetic resonance imaging (MRI) data from 24 healthy controls (HC), 27 patients with cognitively normal Parkinson’s disease (PD-CN), and 33 Parkinson's disease patients with mild cognitive impairment (PD-MCI) were analyzed. Segmentation methods were compared in HC to assess their effect on volume estimates. Group comparisons were then conducted separately for each method to evaluate sensitivity in detecting disease-related volumetric differences. Finally, nuclei with significant group effects in joint segmentation were tested for associations with Addenbrooke’s Cognitive Examination-Revised (ACE-R) scores. Joint segmentation yielded systematically lower thalamic volume estimates than the structural method, with significant differences across hemispheres and nuclei in HC. Group-wise analyses revealed that joint segmentation, but not structural segmentation, detected significant atrophy in the right thalamus of PD-MCI patients. At the nuclei group level, joint segmentation showed greater sensitivity, identifying bilateral anterolateral and posterior nuclei as significantly reduced in PD-MCI relative to HC. Moreover, volumes of these nuclei correlated positively with ACE-R scores. These results highlight that methodological choices critically shape the detection of thalamic pathology in PD, suggesting that incorporating diffusion MRI into segmentation may improve sensitivity to cognitively relevant subnuclear changes and support early diagnosis and disease monitoring.

**Supplementary Information:**

The online version contains supplementary material available at https://doi.org/10.1007/s10548-026-01226-2.

## Introduction

The thalamus, a critical subcortical structure, has long been regarded as a sensory and motor relay station. However, it is now increasingly recognized as a dynamic integrative hub that facilitates communication across widespread cortical and subcortical regions. While it plays a central role in processing and relaying sensorimotor information, a growing body of evidence highlights its involvement in a broad spectrum of neural functions (Ward 2013).

The thalamus comprises multiple distinct nuclei, each characterized by specialized patterns of connectivity, functional specificity, and region-dependent properties (Guillery 1995). These nuclei differ markedly in their structural and physiological properties and are critically implicated in both normal brain functioning and the pathophysiology of various neurodegenerative diseases (Braak et al. 2004; Hari et al. 2024). Functionally, thalamic nuclei are engaged in a wide spectrum of neural processes including pain modulation (Ab Aziz and Ahmad 2006), the modulation of arousal and sleep-wake cycles (Pace-Schott and Hobson 2002), memory (Aggleton et al. 2010; Flanagan et al. 2025), language processing (Barbas et al. 2013), attention regulation (Wright et al. 2015), and executive function (Wolff and Halassa 2024), thereby underscoring the thalamus’s fundamental role in both sensorimotor integration and complex cognitive operations. Given its complex internal architecture and central role in diverse functional circuits, the accurate identification and segmentation of thalamic nuclei are essential for understanding disease mechanisms and monitoring the progression of neurodegenerative conditions.

Accurate segmentation of thalamic nuclei remains a significant challenge due to their small size, limited inter-nuclear contrast, and close spatial proximity within the thalamic complex (Segobin et al. 2024). Conventional structural MRI, although useful for gross anatomical delineation, often lacks the resolution and tissue contrast necessary to distinguish individual nuclei, especially in clinical settings with standard 3T scanners (Iglesias et al. 2018; Su et al. 2019). Even with higher field strength systems (e.g., 7T MRI), where increased spatial resolution is attainable, sufficient contrast between neighboring thalamic subregions is not always achievable (Datta et al. 2021; Segobin et al. 2024). To address these limitations, recent efforts have focused on the development of high-resolution, multi-modal imaging protocols that combine structural, diffusion, and quantitative MRI contrasts (Battistella et al. 2017; Tregidgo et al. 2023). Various segmentation techniques have been developed to delineate thalamic subregions with increasing anatomical and functional precision, facilitating improved mapping of thalamocortical pathways and their alterations in clinical populations (Li et al. 2024). Furthermore, advanced segmentation approaches, such as Bayesian inference models (Tregidgo et al. 2023), deep learning-based methods (Umapathy et al. 2022), and atlas-based joint segmentation frameworks (Iglesias et al. 2018; Su et al. 2019), have significantly improved anatomical accuracy and inter-subject consistency. Tools like FreeSurfer, FSL’s FIRST, and the Morel atlas have been instrumental in thalamic parcellation (Morel et al. 1997, Iglesias et al. 2018), yet each has limitations depending on the resolution, contrast type, and population studied (Williams et al. 2022). Recently, data-driven atlases derived from ultra-high-resolution postmortem histological reconstructions and 7T in vivo MRI acquisitions have demonstrated improved delineation of thalamic substructures, underscoring the value of combining histological precision with advanced MRI contrasts for fine-grained thalamic mapping (Segobin et al. 2024; Vidal et al. 2024).

Despite the variety of available segmentation techniques, each method must be systematically validated, particularly in clinically acquired datasets where imaging parameters often deviate from standardized research protocols. While many publicly available brain imaging datasets have enabled the development and testing of segmentation pipelines under ideal conditions, clinical scans often feature lower resolution, increased noise, and parameter heterogeneity. As such, it remains an open question whether advanced segmentation methods retain their performance and diagnostic utility when applied to real-world clinical data.

In this study, we aimed to evaluate and compare two thalamic segmentation methods: the widely used structural segmentation implemented in FreeSurfer (Iglesias et al. 2018), and a joint segmentation approach that incorporates diffusion tensor imaging (DTI) to enhance anatomical boundary delineation (Tregidgo et al. 2023). The latter was selected because it enables direct comparison between structural-only and diffusion-informed segmentation within the same FreeSurfer-based framework, thereby minimizing methodological variability between approaches. The inclusion of DTI-derived information has the potential to improve the segmentation of thalamic nuclei by leveraging microstructural and connectivity-based cues, which are particularly relevant for distinguishing functionally distinct subregions.

Our central hypothesis is that joint segmentation leveraging DTI data will yield superior anatomical fidelity and improved sensitivity to disease-related changes compared to conventional structural segmentation alone. Specifically, we seek to determine whether the integration of DTI enhances the detection of thalamic alterations in clinical populations and provides added diagnostic value beyond what can be achieved with T1-weighted imaging alone. To test this hypothesis, we utilized neuroimaging data from individuals diagnosed with Parkinson’s disease (PD) and healthy controls (HC). PD was selected as a clinical model due to its well-documented impact on thalamic circuits involved in both motor and cognitive domains. To further investigate the spectrum of cognitive involvement in PD, the patient group was stratified into cognitively normal and mild cognitive impairment subgroups, enabling assessment of whether joint segmentation can detect thalamic alterations associated with varying levels of cognitive decline.

## Materials and Methods

### Participants

A total of 84 participants aged between 45 and 81 years were recruited, comprising 60 patients with idiopathic Parkinson’s disease, including 27 with cognitively normal Parkinson’s disease (PD-CN) and 33 with Parkinson’s disease with mild cognitive impairment (PD-MCI), and 24 neurologically HC. All PD diagnoses were established based on the UK Parkinson’s Disease Society Brain Bank clinical diagnostic criteria (Hughes et al. 1992). All PD patients were recruited through the Movement Disorders Unit of the Neurology Department at the Istanbul University Faculty of Medicine. Written informed consent was obtained from all participants prior to inclusion in the study. Clinical, neuropsychological, and neuroimaging evaluations were carried out while patients were in the ON state, having taken their routine antiparkinsonian medications. Depressive symptoms were assessed in all participants using the Geriatric Depression Scale (GDS) (Yesavage et al. 1982). The dopaminergic treatment dosage was calculated using the Levodopa Equivalent Daily Dose (LEDD) formula as described by Tomlinson et al. (2010). Motor symptoms of PD patients were assessed using the Unified Parkinson’s Disease Rating Scale Part III (UPDRS-III) (Goetz et al. 2008). Global cognitive functioning was assessed in all participants using the Mini-Mental State Examination (MMSE) (Folstein et al. 1975) and the Addenbrooke’s Cognitive Examination–Revised (ACE-R) (Mioshi et al. 2006). PD-MCI was classified using the Level I criteria by Litvan et al. (2012), with an ACE-R cut-off score of ≤ 83 based on normative data from Uysal-Cantürk et al. (2018). Participants were excluded based on the following criteria: (1) a prior history of stroke or head trauma; (2) the presence of notable structural brain anomalies apart from mild white matter hyperintensities observed on FLAIR images; (3) neurological comorbidities; (4) severe psychiatric or systemic diseases or any unstable medical condition that could potentially interfere with clinical or imaging assessments; (5) a Hoehn and Yahr (HY) score > III (Hoehn and Yahr 1967); (6) MRI incompatibilities (e.g., metallic implants, pacemakers, or severe claustrophobia).

### MRI Acquisition

MRI data were acquired at Hulusi Behçet Life Sciences Research Laboratory, Istanbul University using 3 Tesla MRI system (Philips, Achieva, Best, The Netherlands) with a 32-channel SENSE head coil. The structural MRI data were acquired using high-resolution 3D T1-weighted anatomical images obtained with a Turbo Field Echo (TFE) sequence. The imaging parameters were as follows: time of repetition (TR) = 8.4 ms, time of echo (TE) = 3.9 ms, isotropic voxel size = 1 mm³, number of slices = 180, field of view (FOV) = 250 × 250 mm, slice thickness = 1 mm (with no gap), flip angle = 8°, and total acquisition time = 5 min and 55 s. Additionally, T2-weighted images were acquired using a Turbo Spin Echo (TSE) sequence in the axial plane with the following parameters: TR = 10,243 ms, TE = 80 ms, voxel size = 2 mm³, number of slices = 90, FOV = 240 × 240 mm, slice thickness = 2 mm (with no gap), and total acquisition time = 1 min and 42 s. Diffusion-weighted imaging (DWI) was performed using a single-shot echo-planar imaging (EPI) sequence with 32 gradient directions and one non-diffusion-weighted (b = 0 s/mm²) image acquired in a straight axial plane. The acquisition parameters included TR = 10,538 ms, TE = 86 ms, voxel size = 2 mm³, number of slices = 90, FOV = 240 × 240 mm, slice thickness = 2 mm (with no gap), flip angle = 90°, b-value = 1000 s/mm², and total acquisition time = 7 min and 19 s.

### MRI Data Analysis

Structural MRI data were processed using FreeSurfer (version 7.4.1) software (https://surfer.nmr.mgh.harvard.edu). The standard preprocessing pipeline (including motion correction, skull stripping, Talairach registration, intensity normalization, and subcortical segmentation) was applied to the individual T1-weighted structural MRI data of all participants, and automatic segmentation was performed using the *recon-all* command (Fischl et al. 2002). All segmentations were visually inspected to ensure that processing was successfully completed.

**Fig. 1:**
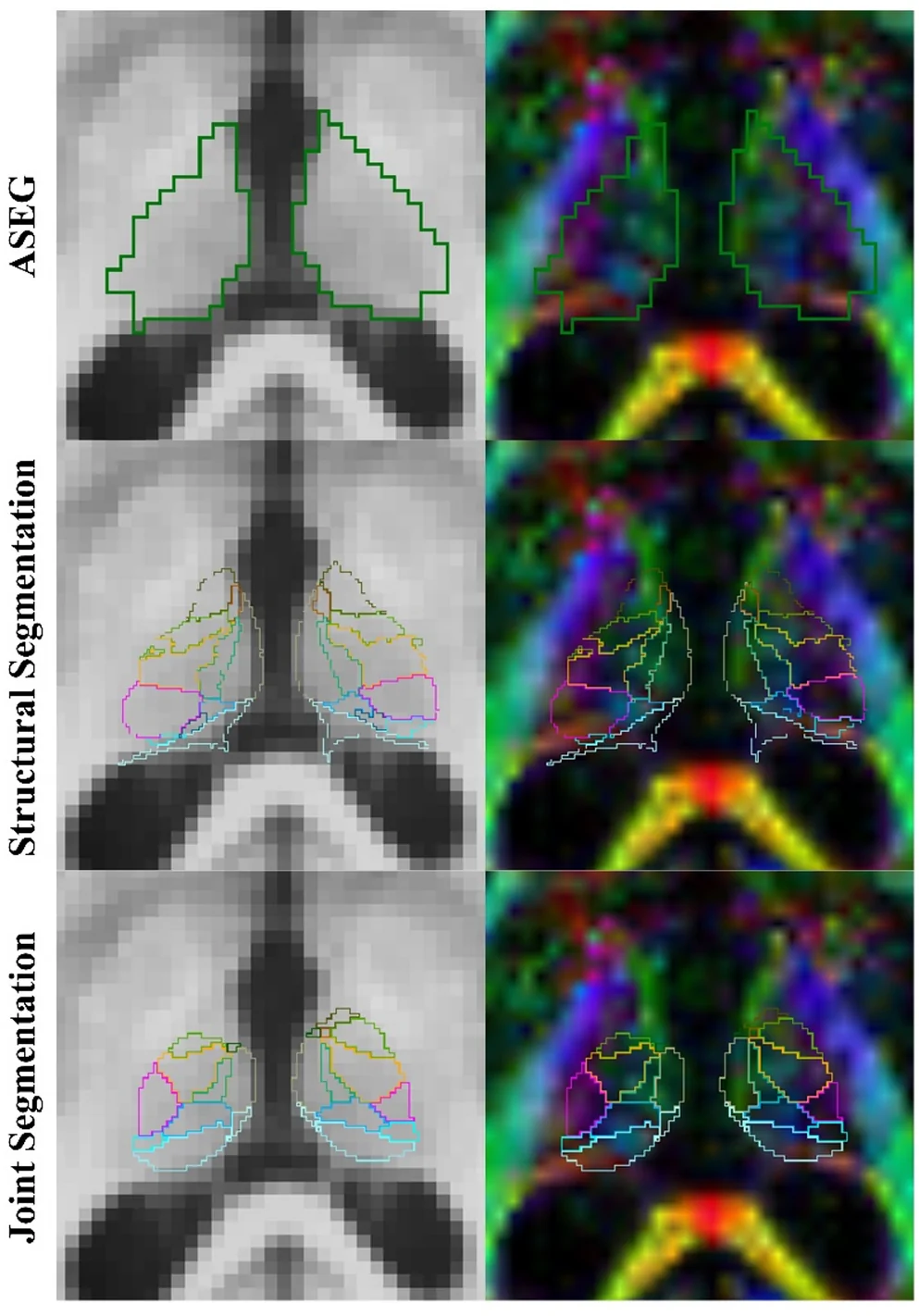
Representative example of thalamic segmentation obtained using the structural and joint segmentation approaches in the same participant. The left column shows segmentations overlaid on T1-weighted structural MRI, while the right column shows segmentations overlaid on color-coded diffusion orientation maps. The top row presents the whole-thalamus segmentation (aseg), the middle row shows the structural-only thalamic nuclei segmentation, and the bottom row shows the diffusion-informed joint segmentation. The incorporation of diffusion-derived information improves boundary delineation

Following this preprocessing stage, thalamic nuclei segmentation was performed using two different approaches. In the first approach, thalamic segmentation relied solely on structural T1-weighted images, using an atlas-based method developed by Iglesias et al. (2018). This approach integrates high-resolution MRI with histological data to delineate 25 distinct thalamic nuclei, enabling the computation of volumetric measurements for each nucleus. In the second approach, a joint (multimodal) segmentation strategy was applied by incorporating diffusion-weighted imaging (DWI) data alongside structural images. DWI data were processed using FMRIB’s Software Library (FSL, version 6.0.3) (Smith et al. 2004). Preprocessing included skull stripping using the Brain Extraction Tool (BET) (Smith 2002), correction of susceptibility-induced distortions with TOPUP (Andersson et al. 2003), and eddy current and motion correction with EDDY (Andersson and Sotiropoulos 2015). Fractional anisotropy (FA) and principal diffusion direction (V1) maps were generated using the DTIFIT module. These diffusion-derived measures were then incorporated into a multimodal thalamic segmentation framework to enhance the delineation of 23 thalamic nuclei (Tregidgo et al. 2023). The method integrates structural and diffusion information within a unified probabilistic model implemented in FreeSurfer, enabling a more precise classification of structural and diffusion properties. Representative examples of the whole-thalamus, structural segmentation, and joint segmentation approaches are shown in Fig. 1.

**Fig. 2:**
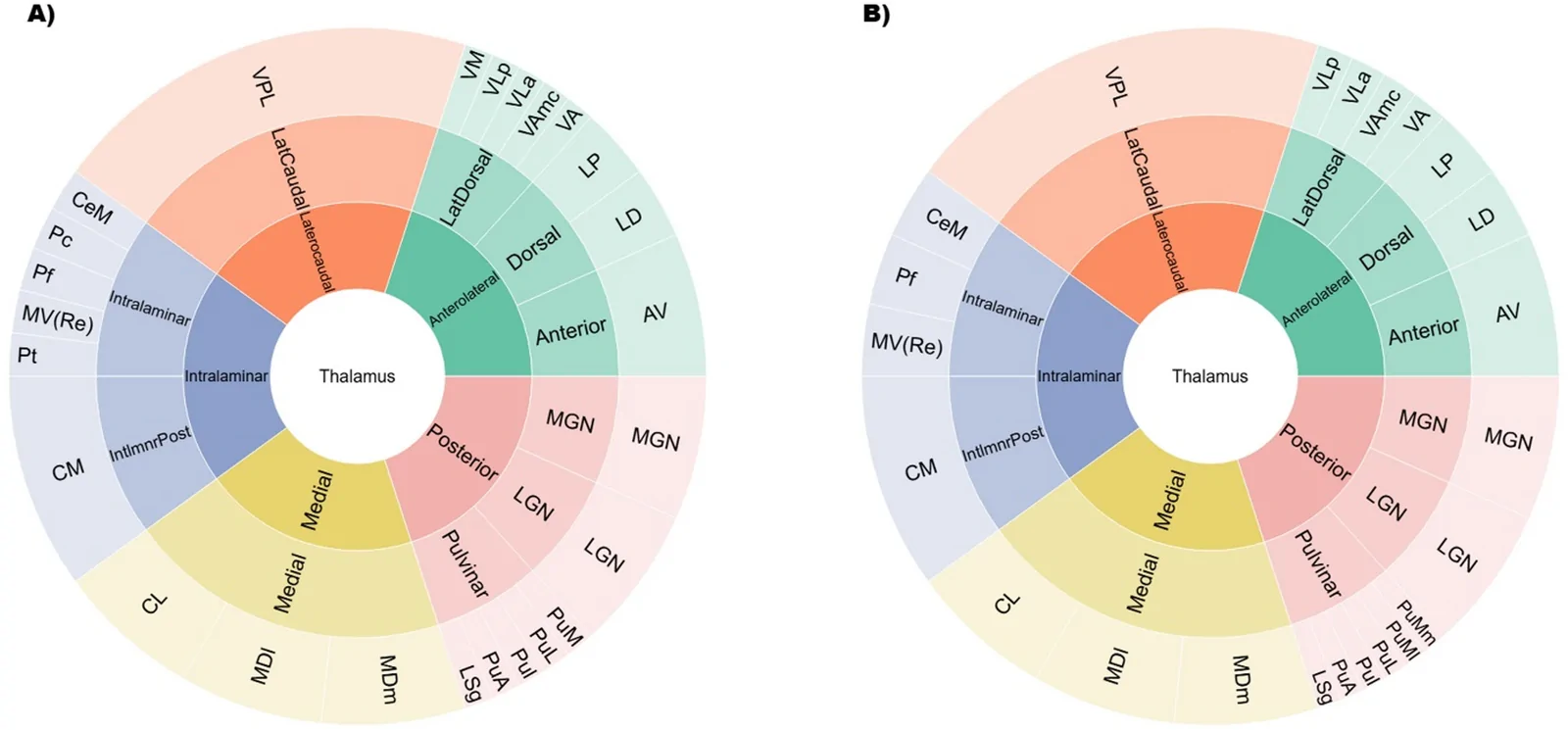
Thalamic nuclei grouping and corresponding labels in structural and joint segmentation methods. (A) Labeled thalamic nuclei (25 nuclei) obtained through the structural segmentation method implemented in FreeSurfer. (B) Labeled thalamic nuclei (23 nuclei) obtained through the joint segmentation method integrating diffusion tensor imaging (DTI) data. The concentric layers represent hierarchical grouping: the innermost ring denotes the main thalamic subdivisions (Antereolateral, Laterocaudal, Intralaminar, Medial, Posterior), the middle ring shows subgroup divisions, and the outer ring lists individual nuclei. Abbreviations: AV=Anteroventral, LD=Laterodorsal, LP=Lateral posterior, VA=Ventral anterior, VAmc=Ventral anterior magnocellular, VLa=Ventral lateral anterior, VLp=Ventral lateral posterior, VM=Ventromedial, VPL=Ventral posterolateral, CeM=Central medial, Pc=Paracentral, Pf=Parafascicular, MV(Re)=Medial ventral (Reuniens), Pt=Paratenial, CM=Centromedian, CL=Central lateral, MDl=Mediodorsal lateral parvocellular, MDm=Mediodorsal medial magnocellular, MGN=Medial geniculate, LGN=Lateral geniculate, PuM=Pulvinar medial, PuMm=Pulvinar medial magnocellular, PuMl=Pulvinar medial parvocellular, PuL=Pulvinar lateral, PuI=Pulvinar inferior, PuA=Pulvinar anterior, LSg=Limitans (suprageniculate)

It should be noted that the structural segmentation identified 25 thalamic nuclei, while the joint segmentation approach delineated 23 nuclei. This discrepancy could affect the comparability of results across methods. To address this, both segmentations were harmonized by grouping the nuclei into five anatomically defined regions—antero-lateral, laterocaudal, intralaminar, medial, and posterior—as proposed by Tregidgo et al. (2023). This grouping ensured an equivalent number of region-based units across methods, allowing for valid comparisons. The regional classification and grouping scheme is illustrated in Fig. 2.

### Statistical Analysis

All statistical analyses were conducted using SPSS 26.0 (IBM, Armonk, N.Y.). Demographic and clinical variables including age, gender, years of education, GDS, MMSE, ACE-R, disease duration, UPDRS-III scores and LEDD were compared across study groups. Age, education and GDS were analyzed using one-way ANOVA, while MMSE and ACE-R scores were evaluated using the Kruskal–Wallis H test due to non-normal distributions. Gender distribution was assessed using the Chi-square test. The disease duration, UPDRS-III scores and LEDD were compared between Parkinson’s disease subgroups using the Mann–Whitney U test.

All thalamic volume values were normalized by dividing by the estimated total intracranial volume (eTIV) to account for individual differences in head size and multiplying the result by 10^6^.

### Comparison of Methods in Healthy Controls

In the HC group, thalamic volumes were obtained using two approaches: structural segmentation and joint segmentation. Volume estimates derived from these methods were compared separately for the left and right hemispheres. To assess differences between the paired measurements, the Wilcoxon signed-rank test was employed. The analysis aimed to determine whether the choice of segmentation method systematically influenced volumetric outcomes. Thereafter, the analyses were extended to the level of thalamic nuclei groups to more precisely investigate how differences between segmentation methods manifested across specific thalamic subregions. To directly compare volumetric differences across segmentation methods, a linear mixed model was implemented. Segmentation method (structural vs. joint) and thalamic nuclei group (antero-lateral, laterocaudal, intralaminar, medial, and posterior) were specified as fixed factors; subject was entered as a random factor. Age, gender, and education were included as covariates. Normalized thalamic volume was specified as the dependent variable. This analysis aimed to assess whether volumetric estimates differed systematically between the two segmentation approaches.

### Comparison of Groups Using Structural and Joint Segmentation

The left and right thalamic volumes obtained from both segmentation approaches (structural and joint), were compared using ANCOVA with age, education, and gender included as covariates. Where appropriate, post hoc pairwise comparisons were performed using Bonferroni-corrected t-tests, with a significance threshold of *α* < 0.05.

To evaluate the ability of each segmentation method to detect group-level differences in thalamic nuclei atrophy, two separate linear mixed models were conducted—one for the structural segmentation data and one for the joint segmentation data. In both models, the diagnostic group (HC, PD-CN and PD-MCI) and the thalamic nuclei group were modeled as fixed factors; the subject was included as a random factor. Age, gender, and education were treated as covariates. Normalized thalamic volume was used as the dependent variable. These models enabled a direct comparison of the sensitivity of each segmentation approach in detecting group-related thalamic volume differences. Following the linear mixed-model analyses, Spearman’s correlation analyses were performed to examine the direction and strength of the associations between ACE-R scores and the thalamic nuclei groups that showed significant differences in the joint segmentation method, combining all study groups. In addition, correlations with disease duration, LEDD, and UPDRS-III scores were examined within the PD cohort only, by combining the PD-CN and PD-MCI groups, as these clinical variables were available only for patients.

## Results

### Demographic and Clinical Results

There were no significant differences in age, gender, education, and GDS, while MMSE and ACE-R scores were significantly different among the groups. The disease duration, UPDRS-III and LEDD were not significantly different between the PD groups (Table 1).

**Table 1:** Demographic and clinical characteristics of groups

	HC (*n* = 24)	PD-CN (*n* = 27)	PD-MCI (*n* = 33)	Comparison
**Age** **(years)**	59.79 ± 7.126	60.37 ± 8.876	63.18 ± 8.353	*F*(2,81) = 1.448^a^, *p* = 0.241
**Gender (F/M)**	9/15	10/17	8/25	*χ*^*2*^(2) = 1.557^b^, *p* = 0.459
**Education (years)**	10.04 ± 4.038	10.15 ± 3.988	8.27 ± 3.660	*F*(2,81) = 2.226^a^, *p* = 0.114
**GDS score**	4.33 ± 3.371	5.74 ± 3.879	6.28 ± 3.970	*F*(2,81) = 1.880^a^, *p* = 0.159
**MMSE score**	29.67 ± 0.816	29.33 ± 0.832	28.03 ± 1.551	***χ***^***2***^**(2) = 25.707**^**c**^, p < 0.001*
**ACE-R total score**	92.58 ± 5.200	89.85 ± 3.687	76.61 ± 5.884	***χ***^***2***^**(2) = 61.101**^**c**^, p < 0.001*
**Disease duration (years)**	N/A	5.37 ± 3.002	6.79 ± 3.998	*U* = 356.50^d^, *p* = 0.183
**UPDRS-III score**	N/A	25.63 ± 10.598	31.27 ± 12.804	*U* = 324.50^d^, *p* = 0.072
**LEDD (mg/day)**	N/A	666.92 ± 353.747	815.56 ± 390.920	*U* = 327.50^d^, *p* = 0.112

HC = Healthy Control, PD-CN = Cognitively normal Parkinson’s Disease, PD-MCI = Parkinson’s Disease with mild cognitive impairment, F = Female, M = Male, GDS = Geriatric Depression Scale, MMSE = Mini-Mental State Examination, ACE-R = Addenbrooke’s Cognitive Examination Revised, LEDD = Levodopa equivalent daily dose (mg/day), N/A = Not Applicable

*p < 0.05

^a^ANOVA, ^b^Chi-square test, ^c^Kruskal-Wallis H test, ^d^Mann-Whitney U test

Pairwise post-hoc comparisons of MMSE and ACE-R scores were conducted using Bonferroni-corrected Mann-Whitney U tests, with a significance threshold of *p* < 0.017. Pairwise comparisons indicated no significant difference in MMSE scores between the HC and PD-CN groups (*p* = 0.042). In contrast, significant differences were observed between the HC and PD-MCI (*p* < 0.001), as well as between the PD-CN and PD-MCI groups (*p* < 0.001) with the PD-MCI group showing lower MMSE scores. Similarly, for ACE-R scores, no significant difference was found between the HC and PD-CN groups (*p* = 0.038), whereas both the HC vs. PD-MCI (*p* < 0.001) and PD-CN vs. PD-MCI (*p* < 0.001) comparisons demonstrated significant differences, again indicating reduced cognitive performance in the PD-MCI group.

### Volumetric Results

In the HC group, thalamic volumes obtained using structural segmentation and joint segmentation methods were compared separately for the right and left hemispheres. The analysis revealed statistically significant differences between the two methods for both the right thalamus (*Z* = 4.286, *p* < 0.001) and the left thalamus (*Z* = 4.257, *p* < 0.001), with the joint segmentation method yielding systematically lower volume estimates.

A linear mixed model analysis was performed to determine differences between the two methods in HCs, specifically focusing on the comparison of thalamic nuclei. The analysis showed a significant main effect of method (*F*(1,437) = 272.002, *p* < 0.001) and nuclei group (*F*(9,437) = 757.160, *p* < 0.001) and interaction of method and nuclei group (*F*(9,437) = 3.847, *p* < 0.001). Post-hoc comparison of the main effect of the method showed that structural segmentation had a significantly higher volume value compared to joint segmentation (*p* < 0.001). The interaction of the method and nuclei group showed that only the left and right intralaminar nuclei group did not differ in the mean volume values obtained by the two methods (Table 2).

**Fig. 3:**
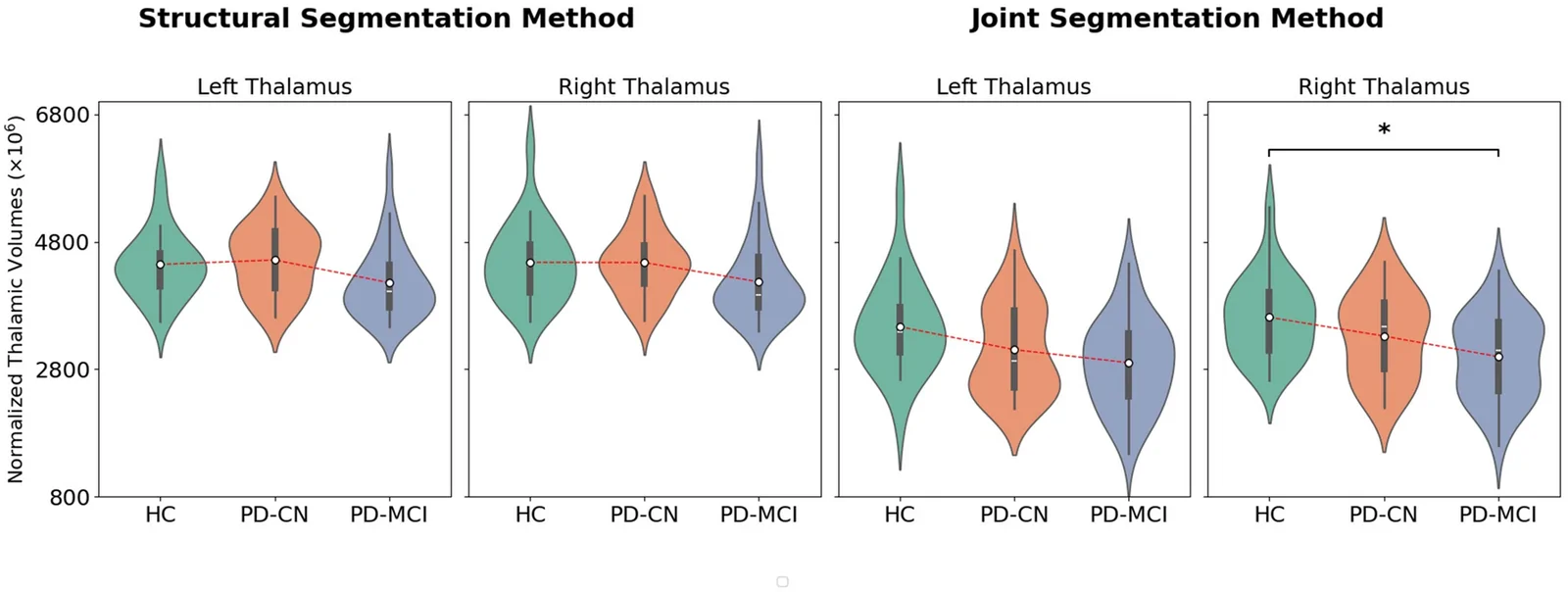
Violin plots showing normalized thalamic volumes for the left and right thalamus across three subject groups: healthy controls (HC), Parkinson’s disease patients with cognitively normal (PD-CN), and with mild cognitive impairment (PD-MCI). Structural segmentation results are shown on the left, and joint segmentation results on the right. A significant volume difference between HC and PD-MCI was observed in the right thalamus using the joint segmentation method. Red lines connect the mean values across groups for visualization. *Significant pairwise difference based on Bonferroni-corrected post hoc tests (*p*_*corr*_ = 0.020).

Group-wise comparisons of normalized thalamic volumes, using ANCOVA, are presented in Table 3. There were no significant differences in left and right thalamus volume for structural segmentation and left thalamus volume for joint segmentation among the groups. However, the right thalamus volume obtained via joint segmentation showed a statistically significant difference between the groups. Post-hoc comparison of the right thalamus volume for joint segmentation showed that the HC group had significantly higher volume compared to the PD-MCI group (Bonferroni-corrected *p* = 0.020). The distribution of the normalized thalamic volume values across groups and segmentation methods is visualized using violin plots in Fig. 3.

The linear mixed model was performed for the structural segmentation method to compare groups. The analysis showed a significant main effect of the nuclei group (*F*(9,729) = 2214.854, *p* < 0.001) and an interaction between group and nuclei group (*F*(18,729) = 1.777, *p* = 0.024). There was no main effect of the group (*F*(2,81) = 1.965, *p* = 0.147).

For the joint segmentation method, the linear mixed-model analysis revealed significant main effects of group (*F*(2,81) = 5.351, *p* = 0.007) and nuclei group (*F*(9,729) = 817.864, *p* < 0.001), as well as a significant interaction between group and nuclei group (*F*(18,729) = 2.143, *p* = 0.004). Post-hoc comparisons showed that the HC group had significantly higher thalamic volumes than the PD-MCI group (*p* = 0.005). In addition, the interaction analysis demonstrated significantly higher volumes in the bilateral anterolateral and posterior nuclei groups in HC compared with PD-MCI (Table 4). Detailed volumetric values for all thalamic nuclei obtained from both segmentation approaches are provided in Supplementary Tables S1 and S2. Spearman’s correlation analysis revealed that all thalamic nuclei groups that showed significant differences in the linear mixed-model analyses were also significantly correlated with ACE-R scores. ACE-R scores were positively correlated with the volumes of the left anterolateral (*r* = 0.306, *p* = 0.005), left posterior (*r* = 0.319, *p* = 0.003), right anterolateral (*r* = 0.346, *p* = 0.001), and right posterior nuclei (*r* = 0.343, *p* = 0.001). Within the PD cohort, no significant correlations were observed between these thalamic nuclei volumes and disease duration, LEDD, or UPDRS-III scores (all *p* > 0.05).

**Table 2:** Linear mixed model results comparing thalamic nuclei volumes between structural and joint segmentation methods in healthy controls (HC)

	Structural Segmentation Method	Joint Segmentation Method	Differences (%)	*p*-value
**Left Thalamus**				
**Antero-Lateral**	1431.0 ± 170.9	1182.8 ± 297.7	17	< 0.001**
**Latero-Caudal**	633.0 ± 64.8	377.3 ± 72.5	40	< 0.001**
**Intralaminar**	266.5 ± 41.3	197.8 ± 63.1	26	0.052
**Medial**	693.3 ± 139.0	471.2 ± 178.1	32	< 0.001**
**Posterior**	1421.4 ± 177.7	1237.7 ± 273.9	13	< 0.001**
**Right Thalamus**				
**Antero-Lateral**	1441.4 ± 206.5	1241.9 ± 275.1	14	< 0.001**
**Latero-Caudal**	579.1 ± 78.2	373.7 ± 73.6	35	< 0.001**
**Intralaminar**	267.5 ± 47.1	207.0 ± 62.9	23	0.087
**Medial**	701.0 ± 149.0	464.7 ± 164.6	34	< 0.001**
**Posterior**	1487.9 ± 192.5	1326.3 ± 192.1	11	< 0.001**

All values represent mean ± SD of normalized thalamic volumes (volume/eTIV × 10⁶)

**p < 0.001

**Table 3:** Group-wise comparisons of normalized thalamic volumes using ANCOVA, controlling for age, gender, and years of education as covariates

		HC	PD-CN	PD-MCI	ANCOVA
**Structural Segmentation Method**	**Left Thalamus**	4445.2 ± 528.5	4514.2 ± 530.1	4157.9 ± 560.2	*F* = 1.371, *p* = 0.260
	**Right Thalamus**	4476.9 ± 613.0	4473.1 ± 515.5	4171.5 ± 604.7	*F* = 0.635, *p* = 0.553
**Joint Segmentation Method**	**Left Thalamus**	3466.8 ± 741.3	3106.8 ± 709.1	2902.2 ± 708.1	*F* = 2.623, *p* = 0.079
	**Right Thalamus**	3613.6 ± 634.2	3320.5 ± 667.7	2996.8 ± 666.3	***F* = 3.933,** p = 0.024*

All values represent mean ± SD of normalized thalamic volumes (volume/eTIV × 10⁶)

HC = Healthy Control, PD-CN = Cognitively normal Parkinson’s Disease, PD-MCI = Parkinson’s Disease with mild cognitive impairment

*p < 0.05

**Table 4:** Results of the linear mixed model comparing normalized thalamic volumes across groups and thalamic nuclei using the joint segmentation method

	HC	PD-CN	PD-MCI	*p*-value		
				HC vs. PD-CN	HC vs. PD-MCI	PD-CN vs. PD-MCI
**Left Thalamus**						
**Antero-Lateral**	1182.8 ± 297.7	1067.8 ± 283.1	977.0 ± 279.2	0.092	**< 0.001**	0.193
**Latero-Caudal**	377.3 ± 72.5	349.4 ± 96.0	314.5 ± 90.3	1.000	0.646	1.000
**Intralaminar**	197.8 ± 63.1	162.2 ± 55.1	165.8 ± 45.5	1.000	1.000	1.000
**Medial**	471.2 ± 178.1	413.0 ± 142.3	365.0 ± 150.3	0.815	0.110	0.984
**Posterior**	1237.7 ± 273.9	1114.4 ± 301.0	1079.9 ± 268.2	0.061	**0.006**	1.000
**Right Thalamus**						
**Antero-Lateral**	1241.9 ± 275.1	1137.4 ± 273.1	1003.3 ± 267.8	0.147	**< 0.001**	**0.019**
**Latero-Caudal**	373.7 ± 73.6	370.4 ± 97.4	325.3 ± 86.5	1.000	1.000	1.000
**Intralaminar**	207.0 ± 62.9	174.6 ± 50.0	163.0 ± 46.2	1.000	1.000	1.000
**Medial**	464.7 ± 164.6	417.7 ± 126.6	362.7 ± 148.0	1.000	0.134	0.785
**Posterior**	1326.3 ± 192.1	1220.3 ± 265.0	1142.5 ± 231.0	0.138	**0.001**	0.337

Bold values indicate statistically significant results (*p* < 0.05)

All values represent mean ± SD of normalized thalamic volumes (volume/eTIV × 10⁶)

HC = Healthy Control, PD-CN = Cognitively normal Parkinson’s Disease, PD-MCI = Parkinson’s Disease with mild cognitive impairment

## Discussion

In this study, volumetric analyses of thalamic nuclei groups were conducted using two segmentation approaches: traditional structural segmentation based on T1-weighted imaging, and a joint segmentation method that integrates both structural and diffusion MRI data. In the HC group, volume estimates derived from structural segmentation were consistently higher than those obtained from joint segmentation across all thalamic nuclei groups, with the exception of the intralaminar nuclei, where the difference did not reach statistical significance.

The structural segmentation method developed by Iglesias et al. (2018) has demonstrated robustness and applicability, leveraging a probabilistic atlas to enable accurate delineation of thalamic nuclei. Built from a multimodal dataset, including ex-vivo and in-vivo MRI, and histology, the deformable atlas adapts to individual anatomical variability. Its reliance on Bayesian inference and voxel-wise intensity likelihoods enables refined, subject-specific delineation of thalamic nuclei without the need for specialized imaging sequences. These features make it particularly suitable for application across diverse neuroimaging datasets. Nevertheless, this structural segmentation method based on image intensity is known to have limitations, particularly in regions with low tissue contrast, such as the lateral thalamus near the internal capsule, where it may overestimate volumes due to partial volume effects (Tregidgo et al. 2023). In the present study, structural segmentation consistently yielded higher volume estimates compared to the diffusion-informed approach, indicating a systematic difference between methods. In the absence of a definitive gold standard, this discrepancy may reflect either overestimation by the structural method or more conservative delineation by the joint segmentation approach. These findings should therefore be interpreted with caution, highlighting the influence of methodological differences on volumetric estimates.

In order to evaluate the relative sensitivity of different segmentation methods in detecting disease-related volumetric differences, we compared structural and joint thalamic segmentation approaches across clinical groups (HC, PD-CN, and PD-MCI). To ensure the validity of these comparisons, potential confounding factors were carefully controlled. In addition to demographic characteristics such as age, gender, and education, disease-related variables that might influence volumetric outcomes were also considered. Demographic and disease-related variables were well matched across groups, and cognitive stratification was confirmed by lower MMSE and ACE-R scores in the PD-MCI group. The absence of differences in disease duration, UPDRS-III, and LEDD between PD-CN and PD-MCI suggests that the observed volumetric alterations may be related to the cognitive status of PD patients rather than disease duration.

When examining total thalamic volumes, the segmentation method had a marked impact. The joint segmentation approach identified significant group differences in the right thalamus, with PD-MCI showing reduced volumes compared with HC. In addition, a descending volumetric gradient was evident across the cognitive spectrum (HC > PD-CN > PD-MCI), indicating that diffusion-informed segmentation may be more sensitive to subtle, progressive neurodegenerative changes. No such effects were observed with structural segmentation, underscoring its limited sensitivity in early disease stages. The literature on thalamic alterations in PD remains inconsistent, with some studies reporting minimal or no volumetric differences (McKeown et al. 2008; Garg et al. 2015), while others describe pronounced atrophy in PD-MCI or PD (Mak et al. 2014; Foo et al. 2017; Vasconcellos et al. 2018; Beheshti et al. 2024), and others reporting heterogeneous or stage-dependent patterns (Chen et al. 2020; Li et al. 2020; Sivaranjini and Sujatha 2021). These discrepancies likely reflect differences in methodology, segmentation approach, cohort size, and cognitive stratification.

At the subnuclear level, linear mixed-model analyses further emphasized the advantage of the joint segmentation method. While structural segmentation revealed no significant group differences, the joint segmentation method detected pronounced atrophy in PD-MCI, particularly in the bilateral anterolateral and posterior nuclei. The anterolateral nuclei, including the ventral anterior and ventral lateral subdivisions, are key nodes in basal ganglia–thalamo–cortical circuits that connect to premotor and prefrontal cortices, supporting executive functions such as working memory and attentional control (Middleton and Strick 2000; Haber and Calzavara 2009; Aggleton et al. 2010). In contrast, the posterior nuclei, encompassing the pulvinar and ventral posterior regions, are heavily interconnected with parietal and occipital cortices, playing essential roles in visuospatial processing and sensory integration (Shipp 2003; Sherman 2007; Saalmann and Kastner 2011). This pattern of volume loss aligns well with the executive dysfunction and visuospatial impairments commonly observed in PD-MCI patients, reflecting the disruption of these specific thalamo-cortical networks. Notably, a significant reduction in the right anterolateral nucleus was observed in PD-MCI compared with PD-CN, indicating that this joint segmentation technique can detect localized subnuclear atrophy prior to the onset of overt dementia. A previous study using structural segmentation reported volume reductions in PD-MCI within the bilateral lateral geniculate, right mediodorsal (medial part), and right anterior pulvinar nuclei, but found no correlations with cognition (Seo et al. 2025). In contrast, our results localized atrophy to the anterolateral and posterior nuclei and demonstrated significant associations with ACE-R scores. The positive correlations between volumetric reductions in these thalamic nuclei and ACE-R scores underscore the functional relevance of these subnuclear regions within thalamo-cortical loops that support cognitive processes, suggesting that atrophy in these nuclei may be closely related to the degree of cognitive impairment in PD-MCI.

Overall, this study demonstrates that the segmentation method influences the detection of thalamic and subnuclear volume changes in PD. The diffusion-informed joint segmentation approach might be more sensitive than structural segmentation for identifying early, cognitively relevant atrophy, particularly in the whole thalamus and in the nuclei level. These results highlight the importance of methodological choices in neuroimaging research and suggest that incorporating diffusion MRI into segmentation protocols may improve the detection of neurodegenerative changes and facilitate the differentiation of clinical subgroups in PD, thereby contributing to the establishment of a consensus in the literature.

### Limitations

This study has methodological and interpretative limitations that should be carefully considered when evaluating the findings. First, although the sample size was adequate to detect statistically significant group differences, validation in larger and independent cohorts is necessary to confirm the generalizability of the results. Besides, the cross-sectional design limits the ability to assess whether the sensitivity of different methods to structural changes varies across disease progression; longitudinal studies are needed to determine these temporal dynamics in PD.

## Conclusion

In summary, our findings indicate that the method used to segment the thalamus can influence the detection of structural changes associated with cognitive impairment in Parkinson’s disease. By integrating diffusion MRI into segmentation, the joint approach demonstrated superior sensitivity in identifying both global and subnuclear atrophy compared with conventional structural segmentation. These results underscore the need for careful methodological consideration in neuroimaging studies and suggest that diffusion-informed segmentation may provide a more reliable biomarker for early cognitive decline in PD, with potential implications for diagnosis, prognosis, and therapeutic monitoring.

## Supplementary Information

Below is the link to the electronic supplementary material.

## Data Availability

No datasets were generated or analysed during the current study.
