# Supplementary material for: Diffusion-Informed Joint Segmentation Enhances Detection of Thalamic Atrophy in Parkinson’s Disease
Source: Brain Topogr. 2026 Jun 30;39(5):74. doi: 10.1007/s10548-026-01226-2 (PMC13319433; doi:10.1007/s10548-026-01226-2)
Supplement: Supplementary file 1 — Supplementary Material 1 [file 10548_2026_1226_MOESM1_ESM.docx]

**Table S1** Structural segmentation-derived thalamic nuclei volumes across study groups

| **Structural Segmentation Method** | | **Left Thalamus**  **(Mean ± SD)** | | | **Right Thalamus**  **(Mean ± SD)** | | |
| --- | --- | --- | --- | --- | --- | --- | --- |
|  |  | **HC** | **PD-CN** | **PD-MCI** | **HC** | **PD-CN** | **PD-MCI** |
| **Antero-Lateral** | **AV** | 86.6 ± 17.6 | 86.7 ± 15.2 | 75.4 ± 15.0 | 93.4 ± 19.5 | 92.6 ± 12.6 | 86.5 ± 18.2 |
|  | **LD** | 17.5 ± 8.1 | 15.7 ± 6.1 | 12.3 ± 6.0 | 16.6 ± 7.9 | 14.4 ± 6.2 | 12.7 ± 7.2 |
|  | **LP** | 77.4 ± 12.7 | 75.7 ± 18.2 | 68.8 ± 17.7 | 75.3 ± 14.1 | 69.3 ± 14.6 | 69.3 ± 15.2 |
|  | **VA** | 273.6 ± 38.2 | 274.0 ± 40.9 | 249.5 ± 38.2 | 277.4 ± 47.4 | 274.5 ± 34.3 | 249.9 ± 42.4 |
|  | **VAmc** | 20.3 ± 3.0 | 21.8 ± 3.3 | 19.2 ± 3.6 | 21.5 ± 4.4 | 22.2 ± 2.5 | 19.9 ± 3.5 |
|  | **VLa** | 404.3 ± 48.0 | 413.5 ± 58.2 | 385.4 ± 57.7 | 411.7 ± 59.2 | 415.0 ± 49.2 | 386.8 ± 55.6 |
|  | **VLp** | 535.7 ± 62.9 | 550.0 ± 74.6 | 512.1 ± 74.2 | 530.6 ± 68.8 | 540.7 ± 63.9 | 508.3 ± 71.0 |
|  | **VM** | 15.7 ± 2.6 | 15.8 ± 3.3 | 14.2 ± 3.6 | 14.9 ± 3.7 | 15.2 ± 2.6 | 13.8 ± 3.0 |
| **Latero-Caudal** | **VPL** | 633.0 ± 64.8 | 631.8 ± 72.6 | 591.2 ± 104.3 | 579.1 ± 78.2 | 603.6 ± 76.5 | 563.6 ± 81.6 |
| **Intralaminar** | **CeM** | 44.8 ± 11.9 | 45.7 ± 8.8 | 41.0 ± 9.9 | 45.9 ± 13.6 | 46.0 ± 8.5 | 41.4 ± 11.0 |
|  | **Pc** | 2.4 ± 0.5 | 2.5 ± 0.4 | 2.2 ± 0.5 | 2.9 ± 0.6 | 2.9 ± 0.4 | 2.6 ± 0.6 |
|  | **Pf** | 39.4 ± 5.2 | 39.9 ± 6.2 | 38.3 ± 6.1 | 42.1 ± 6.8 | 41.9 ± 6.7 | 40.1 ± 6.8 |
|  | **MV(Re)** | 8.6 ± 3.5 | 8.6 ± 2.3 | 7.4 ± 2.3 | 9.3 ± 3.8 | 8.8 ± 2.4 | 7.7 ± 2.8 |
|  | **Pt** | 4.8 ± 0.8 | 4.7 ± 0.6 | 4.6 ± 0.9 | 4.8 ± 0.9 | 4.9 ± 0.7 | 4.6 ± 0.7 |
|  | **CM** | 166.5 ± 24.0 | 171.3 ± 23.7 | 161.7 ± 23.8 | 162.5 ± 25.0 | 167.9 ± 22.9 | 159.6 ± 25.9 |
| **Medial** | **CL** | 20.4 ± 5.1 | 20.9 ± 4.0 | 18.4 ± 4.2 | 20.7 ± 5.6 | 20.9 ± 3.9 | 19.7 ± 5.2 |
|  | **MDl** | 181.9 ± 31.9 | 177.6 ± 22.2 | 165.2 ± 20.2 | 184.3 ± 37.9 | 177.4 ± 24.8 | 169.7 ± 33.3 |
|  | **MDm** | 491.0 ± 105.2 | 506.5 ± 70.5 | 448.6 ± 70.5 | 496.0 ± 109.5 | 498.0 ± 68.9 | 454.5 ± 92.0 |
| **Posterior** | **MGN** | 73.8 ± 13.5 | 73.8 ± 11.9 | 71.0 ± 13.9 | 74.8 ± 15.0 | 76.5 ± 13.8 | 75.1 ± 12.9 |
|  | **LGN** | 171.9 ± 27.1 | 167.9 ± 33.7 | 148.1 ± 33.8 | 166.5 ± 25.8 | 157.8 ± 31.7 | 144.6 ± 28.5 |
|  | **PuM** | 728.8 ± 95.7 | 760.2 ± 97.9 | 693.8 ± 93.7 | 770.6 ± 106.9 | 760.9 ± 110.2 | 704.1 ± 112.1 |
|  | **PuL** | 121.1 ± 17.1 | 120.8 ± 19.7 | 121.6 ± 19.1 | 136.7 ± 21.8 | 127.8 ± 16.4 | 127.6 ± 25.6 |
|  | **PuI** | 161.5 ± 27.0 | 163.4 ± 27.1 | 151.2 ± 22.7 | 175.2 ± 25.0 | 171.2 ± 33.8 | 155.7 ± 30.1 |
|  | **PuA** | 146.7 ± 20.0 | 147.7 ± 21.0 | 139.0 ± 18.9 | 149.3 ± 23.2 | 146.5 ± 19.6 | 138.7 ± 22.6 |
|  | **LSg** | 17.5 ± 4.8 | 17.6 ± 4.9 | 17.5 ± 3.7 | 14.8 ± 3.9 | 16.2 ± 4.7 | 15.0 ± 3.6 |
| All values represent mean ± SD of normalized thalamic volumes (volume/eTIV × 10⁶).  HC=Healthy Control, PD-CN=Cognitively normal Parkinson’s Disease, PD-MCI=Parkinson’s Disease with mild cognitive impairment, AV=Anteroventral, LD=Laterodorsal, LP=Lateral posterior, VA=Ventral anterior, VAmc=Ventral anterior magnocellular, VLa=Ventral lateral anterior, VLp=Ventral lateral posterior, VM=Ventromedial, VPL=Ventral posterolateral, CeM=Central medial, Pc=Paracentral, Pf=Parafascicular, MV(Re)=Medial ventral (Reuniens), Pt=Paratenial, CM=Centromedian, CL=Central lateral, MDl=Mediodorsal lateral parvocellular, MDm=Mediodorsal medial magnocellular, MGN=Medial geniculate, LGN=Lateral geniculate, PuM=Pulvinar medial, PuL=Pulvinar lateral, PuI=Pulvinar inferior, PuA=Pulvinar anterior, LSg=Limitans (suprageniculate). | | | | | | | |

**Table S2** Joint segmentation-derived thalamic nuclei volumes across study groups

| **Joint Segmentation Method** | | **Left Thalamus**  **(Mean ± SD)** | | | **Right Thalamus**  **(Mean ± SD)** | | |
| --- | --- | --- | --- | --- | --- | --- | --- |
|  |  | **HC** | **PD-CN** | **PD-MCI** | **HC** | **PD-CN** | **PD-MCI** |
| **Antero-Lateral** | **AV** | 124.5 ± 47.4 | 107.7 ± 44.0 | 84.2 ± 40.9 | 133.6 ± 48.4 | 115.7 ± 45.4 | 96.3 ± 44.3 |
|  | **LD** | 71.7 ± 24.2 | 69.6 ± 20.0 | 66.3 ± 19.3 | 78.4 ± 24.5 | 77.5 ± 16.8 | 73.8 ± 21.6 |
|  | **LP** | 135.2 ± 20.5 | 128.9 ± 17.7 | 118.8 ± 23.9 | 141.0 ± 24.8 | 133.8 ± 22.5 | 125.1 ± 18.3 |
|  | **VA** | 207.0 ± 82.1 | 172.9 ± 76.4 | 135.2 ± 75.4 | 220.3 ± 76.9 | 178.1 ± 83.1 | 132.0 ± 77.1 |
|  | **VAmc** | 12.2 ± 3.9 | 10.7 ± 3.8 | 10.4 ± 2.5 | 12.1 ± 3.5 | 11.1 ± 4.7 | 10.0 ± 3.1 |
|  | **VLa** | 235.2 ± 79.0 | 209.9 ± 66.7 | 204.5 ± 68.5 | 247.3 ± 70.7 | 227.0 ± 68.8 | 205.8 ± 66.2 |
|  | **VLp** | 397.0 ± 91.0 | 368.3 ± 90.1 | 357.6 ± 83.0 | 409.3 ± 81.9 | 394.4 ± 70.8 | 360.3 ± 81.7 |
| **Latero-Caudal** | **VPL** | 377.3 ± 72.5 | 349.4 ± 96.0 | 314.5 ± 90.3 | 373.7 ± 73.6 | 370.4 ± 97.4 | 325.3 ± 86.5 |
| **Intralaminar** | **CeM** | 56.1 ± 21.4 | 47.7 ± 10.5 | 50.5 ± 12.4 | 61.0 ± 23.9 | 48.3 ± 13.1 | 47.3 ± 15.3 |
|  | **Pf** | 15.6 ± 8.2 | 13.1 ± 7.3 | 13.1 ± 5.6 | 16.0 ± 7.8 | 13.0 ± 6.0 | 12.0 ± 5.5 |
|  | **MV(Re)** | 10.2 ± 7.7 | 6.2 ± 4.7 | 5.4 ± 3.4 | 10.4 ± 7.8 | 6.0 ± 5.0 | 5.4 ± 4.0 |
|  | **CM** | 115.9 ± 40.4 | 95.2 ± 42.6 | 96.7 ± 37.2 | 119.6 ± 37.4 | 107.3 ± 38.7 | 98.2 ± 37.0 |
| **Medial** | **CL** | 59.9 ± 19.5 | 56.0 ± 16.7 | 52.4 ± 13.8 | 63.1 ± 18.7 | 59.7 ± 16.5 | 58.4 ± 17.4 |
|  | **MDl** | 130.2 ± 49.5 | 116.1 ± 35.3 | 106.0 ± 39.0 | 131.0 ± 41.3 | 119.7 ± 33.4 | 107.9 ± 39.5 |
|  | **MDm** | 281.1 ± 118.1 | 240.9 ± 101.7 | 206.6 ± 107.3 | 270.6 ± 113.8 | 238.3 ± 92.1 | 196.4 ± 102.8 |
| **Posterior** | **MGN** | 83.1 ± 32.8 | 78.7 ± 35.9 | 78.9 ± 30.8 | 84.0 ± 27.1 | 84.2 ± 32.8 | 71.3 ± 27.6 |
|  | **LGN** | 146.2 ± 74.8 | 134.6 ± 59.4 | 128.4 ± 59.5 | 153.1 ± 43.9 | 134.4 ± 63.7 | 118.1 ± 55.1 |
|  | **PuMm** | 103.0 ± 50.5 | 80.4 ± 37.9 | 77.1 ± 34.0 | 111.1 ± 39.7 | 88.3 ± 28.2 | 85.9 ± 37.0 |
|  | **PuMl** | 520.3 ± 83.0 | 469.3 ± 105.5 | 448.5 ± 93.7 | 549.2 ± 74.1 | 510.1 ± 89.1 | 485.6 ± 94.6 |
|  | **PuL** | 79.1 ± 20.5 | 70.6 ± 22.9 | 66.6 ± 19.1 | 93.2 ± 19.7 | 87.1 ± 21.0 | 84.8 ± 22.2 |
|  | **PuI** | 132.7 ± 35.9 | 132.0 ± 38.8 | 132.2 ± 33.5 | 152.5 ± 24.5 | 148.3 ± 31.8 | 139.8 ± 24.9 |
|  | **PuA** | 163.0 ± 47.7 | 137.8 ± 38.7 | 137.6 ± 32.7 | 171.3 ± 38.4 | 157.4 ± 34.9 | 146.3 ± 30.4 |
|  | **LSg** | 10.3 ± 3.2 | 11.0 ± 3.3 | 10.7 ± 3.2 | 11.9 ± 4.2 | 10.5 ± 3.4 | 10.7 ± 4.1 |
| All values represent mean ± SD of normalized thalamic volumes (volume/eTIV × 10⁶).  HC=Healthy Control, PD-CN=Cognitively normal Parkinson’s Disease, PD-MCI=Parkinson’s Disease with mild cognitive impairment, AV=Anteroventral, LD=Laterodorsal, LP=Lateral posterior, VA=Ventral anterior, VAmc=Ventral anterior magnocellular, VLa=Ventral lateral anterior, VLp=Ventral lateral posterior, VPL=Ventral posterolateral, CeM=Central medial, Pf=Parafascicular, MV(Re)=Medial ventral (Reuniens), CM=Centromedian, CL=Central lateral, MDl=Mediodorsal lateral parvocellular, MDm=Mediodorsal medial magnocellular, MGN=Medial geniculate, LGN=Lateral geniculate, PuMm=Pulvinar medial magnocellular, PuMl=Pulvinar medial parvocellular, PuL=Pulvinar lateral, PuI=Pulvinar inferior, PuA=Pulvinar anterior, LSg=Limitans (suprageniculate). | | | | | | | |
